# Voice-Hearing Across The Continuum: A Phenomenology of Spiritual Voices

**DOI:** 10.1093/schbul/sbac054

**Published:** 2022-06-23

**Authors:** Peter Moseley, Adam Powell, Angela Woods, Charles Fernyhough, Ben Alderson-Day

**Affiliations:** Department of Psychology, Northumbria University, Newcastle upon Tyne, UK; Department of Theology & Religion, Durham University, Durham, UK; Department of English Studies, Durham University, Durham, UK; Department of Psychology, Durham University, Science Laboratories, Durham, UK; Department of Psychology, Durham University, Science Laboratories, Durham, UK

**Keywords:** hallucinations, comparative phenomenology, distress, control

## Abstract

**Background and Hypothesis:**

Voice-hearing in clinical and nonclinical groups has previously been compared using standardized assessments of psychotic experiences. Findings from several studies suggest that nonclinical voice-hearing is distinguished by reduced distress and increased control. However, symptom-rating scales developed for clinical populations may be limited in their ability to elucidate subtle aspects of nonclinical voices. Moreover, such experiences often occur within specific contexts and belief systems, such as spiritualism. We investigated similarities and differences in the phenomenology of clinical voice-hearing and nonclinical voice-hearer (NCVH).

**Study Design:**

We conducted a comparative interdisciplinary study which administered a semi-structured interview to NCVH individuals (*N* = 26) and psychosis patients (*N* = 40). The nonclinical group was recruited from spiritualist communities. We used content analysis and inductive thematic analysis to create a coding frame which was used across both spiritual and patient groups to compare phenomenological features of voice-hearing.

**Study Results:**

The findings were consistent with previous results regarding distress and control. Additionally, in the NCVH group, multiple modalities were often integrated into 1 entity, and there were high levels of associated visual imagery, and subtle differences in the location of voices relating to perceptual boundaries. Most NCVHs reported voices before encountering spiritualism, suggesting that their onset was not solely due to deliberate practice.

**Conclusions:**

Nonclinical spiritual voice-hearing has important similarities and differences to voices in psychosis. Future research should aim to understand how spiritual voice-hearers cultivate and control voice-hearing after its onset, which may inform interventions for people with psychosis with distressing voices.

## Introduction

Alongside featuring in schizophrenia^1^ and other psychiatric disorders,^2^ hearing voices (or “auditory verbal hallucinations”) occur in nonclinical populations, with some individuals reporting recurring but nondistressing experiences.^3,4^ This offers the opportunity to investigate voice-hearing without the confounds of antipsychotic medication use or comorbid symptoms. Previous studies comparing the phenomenological, cognitive, and neural characteristics of voice-hearing in psychosis patients and nonclinical voice-hearers (NCVHs) have used a combination of standardized scales and clinical interviews for assessing hallucinations. Daalman et al^5^ used the Psychotic Symptoms Rating Scale (PSYRATS) and Auditory Hallucination Rating Scale (AHRS) with 111 NCVHs, reporting that nonclinical voice-hearing was more controllable, less frequent, and less negative. Peters et al^6^ showed that individuals in the general population with persistent psychotic-like experiences often experienced hallucinations across more perceptual modalities than individuals with psychosis, a finding observed elsewhere.^7^ Using the number of different semi-structured and self-report questionnaires with a group of self-reported “clairaudient” psychics, Powers et al^8^ reported NCVHs having higher levels of control over voices (ie, initiating or stopping them at will).

While such findings are important, tools developed for clinical assessment may miss more nuanced aspects of these voice-hearing experiences. The phenomenology of voice-hearing can be highly varied and multimodal: semi-structured and open-ended interviews with psychosis patients have shown the complexity and variation of voice-hearing across individuals, in aspects such as personification, agency, and perceived location.^9,10^ Thus, alternative and interdisciplinary approaches are needed to evaluate the phenomenology of voice-hearing properly.^11^

A case in point is the intersection of spiritual and paranormal experiences with NCVH. Although few studies have sought to recruit people with clairaudient experiences explicitly,^8^ such research often involves people with high levels of spiritual and paranormal belief. Sommer et al^12^ reported that 58% of the participating NCVHs believed their voices came from “benevolent spirits.” Ninety-one percent of healthy individuals with psychotic experiences in Peters et al^6^ classed themselves as “spiritual” (compared to 41% of non-voice-hearing controls, and 76.5% of psychosis patients). Assessing such groups using clinical tools may identify apparently comparable levels of hallucination, but may not sufficiently consider the roles of context, belief, and expectation specific to each community.^13^ For example, a recent online open-ended survey with Christians reporting hearing the voice of God noted key differences from voices in psychosis, including personal significance, positive emotions, and occurrence in the context of prayer.^14^

Important questions remain concerning the development of voice-hearing in such groups. Research with NCVHs has frequently reported an earlier age of onset than in psychosis^6,8,12^; however, it is often unclear whether voice-hearing developed before (perhaps leading to) spiritual beliefs, or whether spiritual beliefs emerged first, with people actively cultivating their voice-hearing experiences. As well as being sharply distinguished from voices in psychosis (unlikely to be purposely developed), this issue may be of relevance to neurocognitive models of voice-hearing that foreground expectations and beliefs.^15,16^ One study regarding spiritual beliefs and voice-hearing^17^ indicated that voice-hearing experiences preceded spiritual beliefs. Although qualitative analysis of mediumistic experiences has emphasized the importance of anomalous childhood experiences,^18^ within-sample variability suggests a more complex relationship between beliefs and voice-hearing. Some participants report voice-hearing starting only when they actively engaged with relevant spiritual practices,^17^ consistent with other research emphasizing the practice (“kindling”) of such experiences.^19^

Here, we investigated nonclinical voice-hearing experiences in individuals with spiritualist and related beliefs (eg, mediums, psychics). Within the United Kingdom, spiritualists typically believe in the possibility of communicating with the dead, often receiving messages they believe should be passed on to other people and working as professional mediums to do so. While there is large variability in specific beliefs surrounding spiritualism, one core tenet is that such experiences should be cultivated and controlled. We used an open-ended, semi-structured interview with spiritualist voice-hearers, developed by an interdisciplinary team for use in both clinical and nonclinical populations. Interviews were coded and directly compared to an extant patient dataset.^10^ We aimed to explore similarities and differences with voices in psychosis that may be missed by standardized scales. While the study was primarily descriptive (ie, not hypothesis-driven), based on previous research, we expected spiritual voices to be less negative, more controllable, and more likely to occur across multiple modalities than voices in psychosis. We also aimed to provide data on the cultivation of voices over time, the nature of the messages communicated by the voices, and the levels of personification and social agency associated with them.

## Method

### Participants

Spiritual NCVH (henceforth referred to as the “spiritual” group; *N* = 26, 19 female) were recruited from spiritualist communities across the United Kingdom, via newsletters, social media advertisements, and word-of-mouth. Participants were mostly over 50 (*M* [*SD*] = 58.23 [11.07] years), although ages ranged from 30 to 73 years. Participants were screened via telephone and were invited to take part if they were aged 18–75 years, fluent English speakers, and reported voices occurring at least once a month that did not solely take place within a spiritualist church (in order to ensure other related experiences, eg, “trance” or meditative practices, were not the sole reason for inclusion). Participants were not eligible to take part if they reported a psychiatric or neurological diagnosis, contacting any mental health services in relation to voice experiences, alcohol or drug abuse within the last 3 months, or severe distress in relation to voices, assessed during telephone screening.

The spiritualist group was compared to 40 psychosis patients (female = 17) recruited from Early Intervention in Psychosis (EIP) services, who completed the same interview procedure as part of a prior study.^10^ The patients were on average younger (*M* [*SD*] = 28.70 [9.96]), with an age range of 16–61 years. Procedures were approved by the relevant university ethics committee.

### Materials

#### The Hearing the Voice Phenomenology Interview.

Participants completed a semi-structured interview, comprising 8 open-ended questions about voices, with each followed by prompts to elicit further details if needed (see supplementary material). The interview was designed by a multidisciplinary team, in consultation with experts-by-experience (see Alderson-Day et al^10^ for further information), and administered by one of 2 interviewers trained in clinical and phenomenological interviewing. Questions were purposely broad (“Please could you describe the voice or voice-like experiences you have been having?”), moving to more specific themes as the interview progressed.

#### PSYRATS (Hallucinations Subscale Only).

The PSYRATS was included as a standardized assessment of voice frequency, duration, location, loudness, distress, controllability, and disruption to life, for comparability with previous research. It contains 11 questions with each item scored between 0 and 4.^20^

### Procedure

After consenting, participants undertook the phenomenology interview, followed by the PSYRATS. Sessions took place in participants’ homes or in a quiet university setting, and typically lasted 60–90 min (range 24–111 min). Participants also completed a questionnaire pack and were invited to take part in cognitive testing and neuroimaging (data reported elsewhere^21^). Interviews were recorded and professionally transcribed.

### Analysis

Similarly to previous work,^9,10^ data were analyzed using a mixture of quantitative and qualitative methods. We used content analysis and inductive thematic analysis to create a coding frame used across both groups. The coding frame (see supplementary material) was developed iteratively, involving discussions after each interview, and co-coding a subset of the spiritual interviews (*n* = 5). A number of additional codes were developed specifically for the spiritual group (eg, regarding spiritual beliefs). Disagreements were resolved via discussion, and the remainder of the interviews were coded separately. Interrater reliability was satisfactory (alpha = .7). The same procedure was used for the patient interviews.^10^ We report descriptive statistics across groups for each code, and log ORs for rate differences between groups, inferring a difference when 95% CIs of log ORs do not cross 0.

## Results

Tables 1–3 present code rates for the spiritual and patient groups, alongside log ORs and 95% CIs. For PSYRATS subscale scores, see supplementary material.

**Table 1.** Sensory Qualities, Location, Control, and Development of Voices

Code	Spiritual (%)	Psychosis (%)	Log Odds	95% CI (Low)	95% CI (High)
Visual imagery	100	5.0	6.705^a^	3.628	9.781
Auditory	84.6	92.5	−0.808	−2.395	0.780
Internally located	84.6	62.5	1.194	−0.049	2.437
Egocentric	80.8	70.0	0.588	−0.599	1.775
Thought-like	76.9	52.5	1.104^a^	0	2.207
Externally located	73.1	80.0	−0.388	−1.55	0.775
Felt presence	69.2	52.5	0.711	−0.328	1.749
Multimodal	69.2	27.5	1.780^a^	0.696	2.864
Visual	60.0	75.0	−0.693	−1.767	0.380
Bodily states	53.8	65.0	−0.465	−1.473	0.543
Olfactory	50.0	37.5	0.511	−0.490	1.511
Dissociative	46.2	30.0	0.693	−0.332	1.719
Tactile	46.2	22.5	1.083^a^	0.012	2.153
Gustatory	26.9	0	3.439^a^	0.526	6.352
Nonverbal	26.9	40.0	−0.593	−1.666	0.480
Allocentric	23.1	37.5	−0.693	−1.808	0.421
Boundary	11.5	35.0	−1.418^a^	−2.785	-0.050

*Note*:

^a^95% CIs do not cross 0.

**Table 2.** Affect, Agency, and Content of Voices

Code	Spiritual(%)	Patient (%)	Log Odds	95% CI (Low)	95% CI (High)
Elicit positive emotions	100	35	4.573^a^	1.703	7.443
Voice knowledge	96.2	45	3.42^a^	1.326	5.513
Volitional	92.3	2.5	6.148^a^	3.695	8.602
Nonvolitional	92.3	100	−2.112	−5.19	0.965
Positive/helpful	92.3	42.5	2.787^a^	1.214	4.360
Recurring	92.3	92.5	−0.027	−1.889	1.834
Ability to influence	84.6	27.5	2.674^a^	1.403	3.946
Change in frequency	80.8	70	0.588	−0.599	1.775
Recognizable	76.9	47.5	1.304^a^	0.201	2.407
Change in influence	73.1	12.5	2.944^a^	1.668	4.221
Simple structure	69.2	40	1.216^a^	0.171	2.262
Conversational	65.4	47.5	0.736	−0.283	1.755
Direct address	61.5	82.5	−1.081	−2.216	0.055
Structural change	50	80	−1.386^a^	−2.478	−0.295
First voice traumatic event	42.3	65	−0.929	−1.943	0.084
Companionship	42.3	32.5	0.421	−0.601	1.442
Elicit negative emotions	38.5	100	-4.846^a^	−7.741	−1.952
Commanding	26.9	67.5	−1.729^a^	−2.820	−0.639
Commenting	19.2	45	−1.234^a^	−2.392	−0.077
Character change	11.5	17.5	−0.486	−1.94	0.967
Abusive/violent	11.5	87.5	−3.983^a^	−5.508	−2.458

Note:

^a^95% CIs of log ORs do not cross 0.

**Table 3.** Personification, Narratives Around Voices, and Effects on Relationships

Code	Spiritual (%)	Patient (%)	Log Odds	95% CI (Low)	95% CI (High)
Supernatural narrative	100	20	5.311^a^	2.414	8.209
Internally individualized	100	75	2.904^a^	0.020	5.788
Externally individualized	80.8	50	1.435^a^	0.28	2.591
Minimal personification	61.5	60	0.065	−0.948	1.077
Important to identity	57.7	12.5	2.256^a^	1.038	3.474
Complex personification	38.5	40	−0.065	−1.077	0.948
Agency w/o individuation	38.5	45	−0.269	−1.275	0.737
Self-stigma	30.8	47.5	−0.711	−1.749	0.328
Negative on relationships	26.9	77.5	−2.235^a^	−3.376	−1.094
Biophysical narrative	23.1	25	−0.105	−1.265	1.054
Archetypal	23.1	42.5	−0.902	−2.009	0.205
Family narrative	19.2	22.5	−0.198	−1.424	1.027
Positive on relationships	15.4	5	1.24	−0.537	3.016
Trauma narrative	15.4	25	−0.606	−1.89	0.677
Sleep disruption	15.4	62.5	−2.216^a^	−3.458	−0.973
Idiosyncratic narrative	11.5	32.5	−1.306	−2.679	0.067
Stress narrative	11.5	37.5	−1.526^a^	−2.889	−0.163
Absent agency	11.5	15	−0.302	−1.786	1.181
Suicidality	3.8	50	−3.219^a^	−5.312	−1.126

*Note*:

^a^95% CI of log ORs do not cross 0.

### Modality and Spatiality

The spiritual group was more likely to report experiences in modalities other than auditory, including *gustatory* (not reported at all in the patient group) and *tactile*. While there was no group difference in rates of *olfactory* or *visual* hallucinations, every spiritual participant reported *visual imagery*, often referring to seeing things in the “mind’s eye.” The spiritual group was also more likely to report *multimodal* voices—experiences in different modalities attributed to the same entity as the voice (regardless of simultaneity).

Spiritual voice-hearers also reported more *thought-like* voices (ie, that could be confused with their own thoughts, and not described as having a sound), although the majority also described *auditory* voices (ie, like hearing a physical voice; see box 1). The spiritual group reported broadly similar rates of *internally* vs *externally* located voices to the patient group, with majorities in both groups reporting that voices sometimes occurred both inside and outside the head. In our previous report, we identified that boundary voices (ie, voices experienced at thresholds, such as coming through the wall or door) often occurred in psychosis, whereas these were relatively infrequent in this spiritual group.

Box 1
**. Modality and Imagery**

**Thought-like voices**

I’m getting told information… to relay to the people who are in the church, so the congregation, if you will… and how I get that is I “hear,” and I use the word in inverted commas because it’s not like I’m hearing you, it’s kind of like thoughts or impressions…[William, age 58 years]

**Visual hallucinations**

you know there’s a gentleman here, he’s taller than yourself, [I can] describe his hair, you know, black hair with a parting and like, his clothes, because they show you how they look… they might adjust their attire…So you can see all that, and then you can say, oh hasn’t he got a deep voice, you know … that’s how it works for me. To me it’s real. But I can understand why you’re asking the question, because if it’s real and they’re standing there, why can’t you see them, yeah?’ [Heather, age 69 years]

**Visual imagery**

and then suddenly I just got a, just an image of a baby and you know just in my head, just an image of a baby, and then just a feeling [John, age 30 years]

**Multimodal voices**

So the next thing that normally comes in will be … either who they were or I’ll hear them say their name, and so I may hear the name, for instance, you’ll get a person who’ll say, “it’s OK, I’m Margaret.” OK, who are you? “Well I’m gran.” At the same time as a picture coming in, as she’s showing what she looked like. Then I’d be asking, well OK, give me more details about yourself, things like age, what did you pass from … And the funny thing is, the voice that you get back very much mimics the personality of who they were. [Samuel, age 48 years]

### Control and Change Over Time

The spiritual group reported more *volitional* experiences, as well as being more likely to report the *ability to influence* their voices. Crucially, when discussing development of voices, the spiritual voice-hearers were more likely to describe a *change in influence* over voices, which tended to reflect the development of increased control (see box 2). This was not the case for other changes over time: there was no group difference for *changes in the character* of voices, while the spiritual group reported less *structural change* over time (eg, changes to number of voices).

Box 2
**. Onset, Cultivation, and Control**

**Spontaneous onset**

I think the first time I ever heard a voice was when I was about eighteen, and I thought I heard somebody calling my name, well I was positive I heard somebody calling my name. And I was the only person in the house at the time. And it sounded like it was coming from outside the house. So I went to the front door, nobody there, went to the back door, walked all the way round the house, nothing, came back in and thought, oh that was weird, wasn’t a radio on, there wasn’t TV on, there was nothing, and I heard it again, calling my name, Laura, Laura, [said with urgency]. And I had to go round the house again, outside, opening all the doors, and I thought, well that’s bizarre, dismiss it, ignore it. So that was the first time it ever happened, I didn’t know anything about spirit or anything like that. [Laura, age 56 years]

I think for me, when I very first started, the only thing that would happen was I’d get my name shouted when I was at work, you know, when there was nobody around… and I think for me sort of I almost questioned if it was real… you know there’s no one else heard it… I think in terms of feelings when I first started having experiences, like I said, I didn’t really have many clairaudient experiences, but I sort of had this feeling of presences… And I think initially I was almost a little bit concerned. And sort of thinking, is there something wrong, you know and being honest, was I mental, was there some sort of illness happening? [John, age 30 years]

**Volitional control**

What I do is I kind of take my consciousness out and allow the spirit world to come in basically…However, sometimes I may not even consciously be thinking about anything and certain words will come into my head. For example if I have, like for example, the other day there I wanted to ask spirit a question and I asked them the question and for the last three days I’ve been getting the same answer… How I do it is I have a little mantra I go through, which is, and I just basically say to the spirit world that I am ready to work… [Ruth, age 50 years]

**Spontaneous onset and cultivation**

There was things happening when I was younger that I didn’t understand at the time, which, once I started learning about it, started to make more sense […] I deliberately developed it, well I’m saying deliberately, I kinda got drawn to it, I was brought up as a Catholic, and I got to… typically get to teenage years and you start questioning things. And I found that the Catholic faith created more questions than answers, and I started looking around for something else. And by the time I think I first went, I would have been about … nineteen, twenty, when I started going to spiritualist churches [William, age 58 years]

### Affect, Agency, and Content

The spiritual group was more likely to report voices that contained *positive content*, *elicited positive emotions*, and correspondingly were less likely to report *abusive or* violent, commanding, or *commenting* voices that *elicited negative emotions*. Instead, their voices were more likely to impart *knowledge* they were not aware of, speak in a *simple structure*, and be *recognizable* as the voices of persons from the voice-hearer’s real life. As part of the coding, the interviews were coded for “levels of agency” ^22^ for auditory verbal hallucination. The spiritual group was more likely to report both *externally individuated* voices (ie, voices attributable to an individual/entity familiar to the voice-hearer from the external world) and *internally individuated* voices (voices not attributable to the external world, but rather internally differentiated by the voice-hearer). All spiritual participants reported at least some internally individuated voices, typically due to their beliefs that (often unrecognizable) spirits would speak in order to pass a message to another person. In our psychosis interviews,^10^ we observed that some patients described highly personified voices (*complex personification*), exhibiting their own intentions, emotions, and personality characteristics. This rate was similar in the spiritual group, suggesting that the groups are not distinguished by varying degrees of personification.

### Social Context and Interpretation

Unsurprisingly, the most common narrative applied to voices in the spiritual group was around the *supernatural*, with all participants in that group describing belief in a supernatural explanation. That said, a substantial number also described potential *biophysical* explanations or described the experiences “running in the family,” at a similar rate to the patient group. The spiritual group was less likely to offer *stress* as an explanation than the patients.

The spiritual group was also less likely to say that their voices caused a negative impact on relationships with others; mention disrupted *sleep*; and describe suicidal ideation. A majority of the spiritual group suggested that their voices were an *important part of their identity*, with social aspects of their spiritual beliefs playing an important role.

We also developed a group of codes applicable only to the spiritual group. These indicated that most of the spiritual group reported that their first voice experiences were spontaneous (88.5%) and tended to precede their beliefs in spiritualism (69.2%). Most engaged in ongoing cultivation of the voices (96.2%) and viewed voice-hearing/clairaudience as a learnable skill (76.9%). Finally, most were highly convinced of their spiritual beliefs (80.8%), and most used their voice-hearing as part of a profession (eg, as a medium) (88.5%).

## Discussion

We conducted phenomenological interviews with a group of NCVHs reporting spiritual voices. Despite much overlap in the voice-hearing experience, group differences included variation in control, multimodality, and valence, as well as nuanced differences in descriptions of location.

Consistent with the previous studies^7^ with NCVHs, the spiritual voice-hearers were more likely to report experiences occurring across sensory modalities, being more likely to describe tactile or gustatory experiences, as well as visual imagery associated with voices. A novel finding was that voices themselves were more likely to be multisensory, compared to psychosis: not only did spiritual voice-hearers report experiences co-occurring across modalities, but they were also more likely to attribute them to the same entity (eg, a spirit). Our analysis of interviews also distinguished between experiences such as visual hallucinations and visual imagery. All participants in the spiritual group reported visual imagery in association with voice-hearing, with rates of visual hallucinations comparable to the psychosis group. This highlights an important consideration relating to understanding the experiences of different voice-hearing groups: the language used to describe voices—often shaped by clinicians (in psychosis patients) or peers (in the spiritual group)—can have large effects on interpretations by researchers.^11,23–25^ Many spiritual participants reported visual imagery in their “mind’s eye” that they distinguished from typical visual imagery by its spontaneity and/or alien quality, but which was easily distinguished from external visual perception.

This might raise the worry that spiritual voice-hearers are generally having an experience more akin to imagery than something resembling “true” hallucination, but the majority of spiritual voice-hearers in our study did report some experience of externalized, vivid perceptions which they held to be really occurring. Moreover, voice-hearing in psychosis is often described with “thought-like” qualities (with majorities of both the spiritual and psychosis groups reporting sometimes having thought-like experiences) and is often located inside the head. Traditional categorization into “true” and “pseudo” hallucinations is nowadays thought to be of questionable clinical relevance.^26^ It is unclear whether high rates of visual hallucinations reported in other nonclinical voice-hearing studies may include such reports of imagery. Furthermore, detailed phenomenological research would be needed to understand whether the difference between such experiences is quantitative (eg, amount of perceptual detail) or qualitative.

As reported in previous studies,^8,27–29^ control or influence over voices seems to differentiate clinical and nonclinical groups. Methods by which control was exerted over the experience varied widely between participants, with some describing a process of “tuning in” (typically described as practicing self-directed attention, to put oneself in a “state of mind” to bring on the voices) and others describing a more elaborate process of influencing the voice via dialogue. A key distinction here is between volitional control (ability to bring on or stop voices intentionally), and influence over voices (through other strategies such as engagement or distraction), referred to elsewhere as *direct* and *indirect* control.^27^ The spiritual group reported substantially higher levels of control and influence compared to patients. Consistent with previous research demonstrating that control may increase over time,^30^ nearly 3 quarters of the group reported a *change* in their ability to influence the voices over time—compared to 12.5% of psychosis patients—suggesting that this ability is not always present from the onset of voice-hearing in nonclinical populations and instead can be actively developed; 88.5% of our spiritual group described their voices starting spontaneously, with 69.2% reporting that this was before they had contact with spiritualism itself. Thus, while most of the group (96.2%) reported ongoing cultivation of the voices, and often reported developing influence over time, it seems that spiritual practices mostly do not elicit the actual initial onset of the voices, instead playing a role in honing the experience. This is consistent with previous qualitative analysis suggesting that mediums, and people of Christian faith who report hearing the voice of God,^14^ often discuss nonspiritual anomalous experiences in childhood^18^ which, as adults, are only later interpreted as spiritual in nature. The high prevalence of reported ongoing cultivation of voices, typically within a spiritualist community setting, may support the suggestion that culture has a role to play in shaping how voices are experienced and reported by different groups,^24,31^ possibly affecting reports of multimodality, control, vividness, and distress, and also suggesting that how voices are interpreted and reacted to in early psychosis could predict future functioning. NCVHs in other studies have also reported earlier ages of onset compared to psychosis patients, and childhood experiences such as imaginary companions are increasingly recognized as sharing some underlying cognitive markers.^32^ While others have argued for the importance of spiritual practice, expectation, and belief in the occurrence of voices or hallucination-like experiences,^19^ our data suggest that although the spiritual practices and beliefs reported by this group may play a role in developing control over and influencing the experience, possibly across sensory modalities, for most, they may be less important in initial onset.

It is noteworthy that, despite the rarity of negative or abusive voices in this group, 42.3% of the spiritual group reported some form of traumatic event around the point of initial voice onset. These experiences were often nonviolent events (eg, family separation, bereavement), but also included parental verbal or physical abuse. While it is well established that people with psychosis report heightened rates of adverse childhood experiences,^33^ our data show that this does not necessarily inform negative content of experiences. One possibility is that spiritual practices may alleviate or control negative affective elements of the experience: for example, many spiritual mediums report that they interpret many voices as containing a message meant for other people, which could reduce negative impact and mean that voices are seen as inherently helpful.

Whether initially experienced, or latterly interpreted, as comforting/positive, it is noteworthy that a majority of the spiritual group understood voices as important to their sense of identity. The salience of “voice-hearer” as an identity has been forged in opposition to the “psy” disciplines by those who may otherwise have been labeled “schizophrenic.” It will be important in the future to investigate the relationship between comforting/controllable voices and personal identity as well as whether mental health treatments can avoid the interpellation of patient identities while retaining therapeutic gains. It is also worth mentioning that, for a minority of participants, voices only occurred after contact with (and presumably as a result of) spiritual practices. An important question for future research is to understand differences between the spontaneous and nonspontaneous onset of voices.

As noted by others,^27^ the spiritual group’s ability to exert control over voices may have much to tell us about coping strategies that could be employed by people experiencing distressing voices; for example, the spiritual voice-hearers described using techniques similar to meditation, mindfulness, or voice-dialogue to control or influence voices. However, because the spiritual voice-hearer group reported a higher rate of positive and helpful voices, their experiences may be more amenable to the development of control or influence — that is, positive voices could be constructively engaged with, which may not be the case with more distressing experiences. An alternative position to the aforementioned potential role of cultural differences is that differences in cognition could at least partially underlie differences in control between the groups. A separate study^21^ investigating cognition in this sample suggested that ability to intentionally inhibit intrusive memories may be higher in the spiritual group than in voice-hearing psychosis patients, raising the possibility that inhibitory ability could underlie control over voices.

The issue of location (internally vs externally located voices) has also been discussed in previous research, with mixed findings. Our interviews suggested that the spiritual voice-hearers were no more likely to describe internally located voices. However, one novel finding was that the spiritual group was less likely to describe *boundary* voices—that is, voices located at thresholds such as doors or through walls—compared to the patient group. In psychosis patients, boundary voices seem to be linked to descriptions of uncertainty or anxiety regarding who is speaking; in contrast, the spiritual group, while often not initially knowing the source of a voice, are used to engaging with and interpreting their voices (ie, minimizing uncertainty) and, as discussed, typically develop control and influence over their experiences. It may be that increased engagement with the voices leads to clearer and more certain reports of both the source and location of the voice; alternatively, the “boundary” location of the voice may play a part in the associated distress of some patients (ie, anxiety regarding a voice crossing into their personal space), meaning that such experiences are less likely to be nondistressing and thus deemed “nonclinical.”

There were a number of limitations to the present study. Firstly, although the sample is not small within qualitative research, our design precludes strong statistical inferences, particularly regarding equivalence between groups. Secondly, the spiritualist and patient groups differed on a number of demographic attributes, including education, gender, and age. The 2 groups were recruited in separate studies; we aimed to treat previously published patient data as a reference point, rather than a matched control group. It seems unlikely that key differences between the groups (eg, modality of experiences) could be explained by such group differences. Where differences relating to demographic attributes may exist, they are likely to interact with group status and voice phenomenology in ways that would not be possible to untangle here. Finally, the spiritualist voice-hearer group predominantly represented beliefs and traditions endorsed by spiritualist churches in the United Kingdom (and were specific to our method of recruitment, including requiring participants to report experiences not solely occurring in a spiritualist church), and the sample was of a primarily white British demographic. Furthermore, research should attempt to increase the diversity of both clinical and nonclinical groups recruited into nonclinical voice-hearing research, including investigating spiritual experiences in younger adults or adolescents. It is notable that ethnic minority groups show higher rates of diagnosed psychotic disorders in the United Kingdom and the United States, and recent research with Black psychosis service-users has suggested that interventions should take into account personal beliefs regarding mental health,^34^ which may include different psychosocial and spiritual explanations between ethnic groups.

In summary, using a mixed-methods approach we compared voice phenomenology in a nonclinical, spiritual voice-hearer group to a group of voice-hearing psychosis patients. As well as supporting previous findings regarding low levels of distress and increased control and multimodality in nonclinical groups, we also provided novel evidence of other more subtle differences, including a lower likelihood of experiencing voices coming from perceptual boundaries, and increased integration of modalities into one entity. The majority of participants in the spiritual group reported unusual sensory experiences before encountering spiritualism, suggesting that, while spiritual practices may shape, influence, or increase the frequency of such experiences, they are not typically involved in the initial onset. The mixed-methods approach employed here revealed novel differences between voice phenomenology reported by different groups that might have been missed by standardized clinical assessments (eg, boundary voices, differences between visual imagery and hallucination). Our replication of previous findings (reduced distress and increased control in the spiritual group) using a different methodological approach can increase our confidence in their veracity. Future research should aim to understand more about strategies used to control voices, which could inform interventions for those in distress.

## Supplementary Material

sbac054_suppl_Supplementary_Material

## References

[1] Bauer SM , Schanda H, Karakula H, et al Culture and the prevalence of hallucinations in schizophrenia. Compr Psychiatry. 2011;52(3):319–325. 21497227 10.1016/j.comppsych.2010.06.008

[2] Toh WL , Thomas N, Rossell SL. Auditory verbal hallucinations in bipolar disorder (BD) and major depressive disorder (MDD): a systematic review. J Affect Disord. 2015;184:18–28. 26066781 10.1016/j.jad.2015.05.040

[3] Larøi F , Sommer IE, Blom JD, et al The characteristic features of auditory verbal hallucinations in clinical and nonclinical groups: state-of-the-art overview and future directions. Schizophr Bull. 2012;38(4):724–733. 22499783 10.1093/schbul/sbs061 PMC3406519

[4] Johns LC , Kompus K, Connell M, et al Auditory verbal hallucinations in persons with and without a need for care. Schizophr Bull. 2014;40(suppl 4):S255–S264. 24936085 10.1093/schbul/sbu005 PMC4141313

[5] Daalman K , Boks MPM, Diederen KMJ, et al The same or different? A phenomenological comparison of auditory verbal hallucinations in healthy and psychotic individuals. J Clin Psychiatry. 2011;72(03):320–325. 21450152 10.4088/JCP.09m05797yel

[6] Peters E , Ward T, Jackson M, et al Clinical, socio-demographic and psychological characteristics in individuals with persistent psychotic experiences with and without a “need for care.”. World Psychiatry. 2016;15(1):41–52. 26833608 10.1002/wps.20301 PMC4780307

[7] Toh WL , Thomas N, Robertson M, Rossell SL. Characteristics of non-clinical hallucinations: a mixed-methods analysis of auditory, visual, tactile and olfactory hallucinations in a primary voice-hearing cohort. Psychiatry Res. 2020;289:112987. 32446007 10.1016/j.psychres.2020.112987

[8] Powers AR , Kelley MS, Corlett PR. Varieties of voice-hearing: psychics and the psychosis continuum. Schizophr Bull. 2017;43(1):84–98. 28053132 10.1093/schbul/sbw133 PMC5216860

[9] Woods A , Jones N, Alderson-Day B, Callard F, Fernyhough C. Experiences of hearing voices: analysis of a novel phenomenological survey. Lancet Psychiatry. 2015;2(4):323–331. 26360085 10.1016/S2215-0366(15)00006-1 PMC4580735

[10] Alderson-Day B , Woods A, Moseley P, et al Voice-hearing and personification: characterizing social qualities of auditory verbal hallucinations in early psychosis. Schizophr Bull. 2021;47(1):228–236. 33484268 10.1093/schbul/sbaa095 PMC7824995

[11] Woods A , Jones N, Bernini M, et al Interdisciplinary approaches to the phenomenology of auditory verbal hallucinations. Schizophr Bull. 2014;40(suppl 4):S246–S254. 24903416 10.1093/schbul/sbu003 PMC4141308

[12] Sommer IEC , Daalman K, Rietkerk T, et al Healthy individuals with auditory verbal hallucinations; who are they? Psychiatric assessments of a selected sample of 103 subjects. Schizophr Bull. 2010;36(3):633–641. 18849293 10.1093/schbul/sbn130 PMC2879692

[13] Woods A , Wilkinson S. Appraising appraisals: role of belief in psychotic experiences. Lancet Psychiatry. 2017;4(12):891–892. 29179922 10.1016/S2215-0366(17)30434-0

[14] Cook CCH , Powell A, Alderson-Day B, Woods A. Hearing spiritually significant voices: a phenomenological survey and taxonomy [published online ahead of print December 7, 2020]. Med Humanit. doi: 10.1136/medhum-2020-012021. PMC9411900 33288684

[15] de Lange FP , Heilbron M, Kok P. How do expectations shape perception? Trends Cogn Sci. 2018;22(9):764–779. 30122170 10.1016/j.tics.2018.06.002

[16] Hohwy J. The Predictive Mind. Oxford, UK: Oxford University Press; 2014.

[17] Powell A , Moseley P. When spirits speak: absorption, attribution, and identity among spiritualists who report “clairaudient” voice experiences. Ment Health Relig Cult. 2021;23(10):841–856. doi: 10.1080/13674676.2020.1793310.

[18] Roxburgh EC , Roe CA. Reframing voices and visions using a spiritual model. An interpretative phenomenological analysis of anomalous experiences in mediumship. Ment Health Relig Cult. 2014;17(6):641–653.

[19] Luhrmann TM , Nusbaum H, Thisted R. The absorption hypothesis: learning to hear God in evangelical Christianity. Am Anthropol. 2010;112(1):66–78.

[20] Haddock G , McCarron J, Tarrier N, Faragher EB. Scales to measure dimensions of hallucinations and delusions: the Psychotic Symptom Rating Scales (PSYRATS). Psychol Med. 1999;29(4):879–889. 10473315 10.1017/s0033291799008661

[21] Moseley P , Alderson-Day B, Common S, et al. Continuities and Discontinuities in the Cognitive Mechanisms Associated with Clinical and Non-Clinical Auditory Verbal Hallucinations [published online ahead of print]. Clin Psychol Sci. 2022. doi: 10.1177/21677026211059802. PMC9280701 35846173

[22] Wilkinson S , Bell V. The representation of agents in auditory verbal hallucinations. Mind Lang. 2016;31(1):104–126. 26900201 10.1111/mila.12096 PMC4744949

[23] Luhrmann TM. Diversity within the psychotic continuum. Schizophr Bull. 2017;43(1):27–31. 27872266 10.1093/schbul/sbw137 PMC5216862

[24] Larøi F , Luhrmann TM, Bell V, et al Culture and hallucinations: overview and future directions. Schizophr Bull. 2014;40(suppl 4):S213–S220. 24936082 10.1093/schbul/sbu012 PMC4141319

[25] Collins LC , Semino E, Demjén Z, et al A linguistic approach to the psychosis continuum: (dis)similarities and (dis)continuities in how clinical and non-clinical voice-hearers talk about their voices. Cognit Neuropsychiatry. 2020;5(6):447–465. 10.1080/13546805.2020.1842727 PMC7713671 33158372

[26] Copolov D , Trauer T, Mackinnon A. On the non-significance of internal versus external auditory hallucinations. Schizophr Res. 2004;69(1):1–6. 15145464 10.1016/S0920-9964(03)00092-6

[27] Swyer A , Powers AR. Voluntary control of auditory hallucinations: phenomenology to therapeutic implications. Npj Schizophr. 2020;6(1):1–9. 32753641 10.1038/s41537-020-0106-8 PMC7403299

[28] Honig A , Romme MAJ, Ensink BJ, Escher SC, Pennings MHA, Devries MW. Auditory hallucinations: a comparison between patients and nonpatients. J Nerv Ment Dis. 1998;186(10):646–651. 9788642 10.1097/00005053-199810000-00009

[29] Baumeister D , Sedgwick O, Howes O, Peters E. Auditory verbal hallucinations and continuum models of psychosis: a systematic review of the healthy voice-hearer literature. Clin Psychol Rev. 2017;51:125–141. 27866082 10.1016/j.cpr.2016.10.010 PMC5240854

[30] Mourgues C , Hammer A, Fisher V, et al Measuring voluntary control over hallucinations: the Yale Control Over Perceptual Experiences (COPE) scales. Schizophr Bull. 2022;48(3):673–683. 10.1093/schbul/sbab144 PMC9077437 35089361

[31] Luhrmann TM , Padmavati R, Tharoor H, Osei A. Hearing voices in different cultures: a social kindling hypothesis. Top Cogn Sci. 2015;7(4):646–663. 26349837 10.1111/tops.12158

[32] Fernyhough C , Watson A, Bernini M, Moseley P, Alderson-Day B. Imaginary companions, inner speech, and auditory verbal hallucinations: what are the relations? Front Psychol. 2019;10:1665. 31417448 10.3389/fpsyg.2019.01665 PMC6682647

[33] Varese F , Smeets F, Drukker M, et al Childhood adversities increase the risk of psychosis: a meta-analysis of patient-control, prospective- and cross-sectional cohort studies. Schizophr Bull. 2012;38(4):661–671. 22461484 10.1093/schbul/sbs050 PMC3406538

[34] Bard S , Degnan A, Berry K, Edge D. Exploring the relationships between illness beliefs and psychosis symptoms among Black African and Caribbean people with non-affective psychosis. Psychosis. 2021;13:265–275.

