# Supplementary material for: Voice-Hearing Across The Continuum: A Phenomenology of Spiritual Voices
Source: Schizophr Bull. 2022 Jun 23;48(5):1066–74. doi: 10.1093/schbul/sbac054 (PMC9434432; doi:10.1093/schbul/sbac054)
Supplement: sbac054_suppl_Supplementary_Material [file sbac054_suppl_supplementary_material.docx]

**S1. Hearing the Voice phenomenology interview**

1. **Could you try to describe to me some of the voice (or voice-like) experiences you’ve been having?**

Prompts

- *How, if at all, are these experiences different from your own thoughts?*
- *How, if at all, are these experiences different from hearing the voice of someone who is present in the room?*
- *Are there any other senses (e.g. images, tastes or smells) involved in the experience?*
- *Does it have a location (i.e. does it feel like you can tell where the voice is coming from)?*
- *Number of different voices/identities (if volunteered)*
- *If there is more than one voice, how do you distinguish between them? i.e. do they sound different, do they have different characters, do they say different things?*
- *When was the last time you had this kind of experience?*

1. **Could you tell us a bit about what life was like for you, and how you were feeling, when you first started having these experiences?**

Prompts

- *Do you remember when you first heard a voice? (establish age estimate)*
- *If so, can you describe it?*
- *Was it similar to your recent voice/voice-like experiences?*
- *How were things going at the time?*
- *When did you first seek help or treatment?*

1. **How does it feel when you have the experience?**

Prompts

- *What kinds of moods or emotions are associated with your voices?*
- *Do you know when you are about to experience a voice? If so, how?*
- *Does your body feel different when you experience voices?*
- *Are there particular times or places when the experience is likely to occur?*
- *How do you feel towards your voice? Is this always the case, or does it vary?*

1. **Do your experiences contain messages of any kind?**

Prompts

- *What kinds of things does the voice say?*
- *Are there specific words or sentences that are used? Can you give me any examples?*
- *Do you always understand the message (what is being said)?*
- *How is it expressed? (e.g. friendly, unfriendly, angry, dominant, commanding, supportive, loud, quiet)*
- *How can you tell how it is expressed? Is it the tone of voice, for example?*
- *Is the message ever spiritual or religious?*

1. **Does it feel as though the experiences have their own character or personality?**

Prompts

- *Do you know who they are? If so, how?*
- *Do they remind you of anyone you know or have known?*
- *If so, is it the voice or what they say that reminds you of this person?*
- *Are they always in the same form (e.g. a voice, a vision, a presence?)*
- *Do they mean well?*
- *Do they know things that you do not?*
- *Are they a spiritual being?*

1. **Have your experiences changed at all since they first started?**

Prompts

- *Has it tended to happen more or less over time?*
- *Over time, have you been able to influence your voices? If so, how?*
- *How, if at all, have your voices affected your relations with other people?*

1. **Why do you think these experiences are happening?**

Prompts

- *What do the voices mean to you?*
- *Do you consider hearing voices to be a special ability or skill?*
- *Have they been caused by something that has happened to you, do you think?*
- *How do you think other people view this experience?*
- *Have you developed any ideas about why you have these experiences?*
- *Are you a religious or spiritual person?*
- *Have any of your family members had similar experiences?*

1. **Is there anything else we haven’t talked about yet, but is an important part of your experience?**

Prompts

- *Presence of any other senses or multisensory elements (if not already covered)*
- *Have you also had any other experiences that you think might be linked?*

**S2. Hearing the Voice Phenomenology Interview coding scheme**

*This coding frame is intended to apply to the Hearing the Voice Phenomenology Interview. Codes are applied if there is clear evidence in the transcript of a particular experience or aspect of experience being reported.*

1. **MODALITY**

**A1. Does the participant report ‘auditory’ experiences?**

Does the participant report experiences that are like actually hearing? Note that this does not mean the voices have to be perceived as located externally to the head (i.e., you could have an auditory voice within your head).

**A2. Does the participant report ‘thought-like’ experiences?**

Does the participant report experiences that are described as being similar to their thoughts? Note, again, that this is a separate issue to location. Any mention of confusion/overlapping with own thoughts coded.

**A3. Are the voices multisensory?**

Does the participant seeing, touching, smelling, etc, the voice (or the ‘person/character behind the voice’)? This doesn’t necessarily have to be concurrently with hearing, but there should be clear reference to, for example, seeing the person that is heard (even if at separate time points).

**A4. Does the participant report non-verbal auditory hallucinations?**

For example, hearing music, bangs, clicks. This could also include non-verbal voices (e.g., moaning, screaming with no words).

**A5. Does the participant report visual hallucinations?**

Does the participant report experiences of seeing things – this does not have to be directly related to the voices. This should be specific reference to seeing things ‘out there’ in the world – i.e., externally, not just in the ‘mind’s eye’, for example, seeing a person across the room, seeing shadows out of the corner of your eye.

**A6. Does the participant report visual imagery-like experiences?**

This could include ‘seeing things in my head’, things ‘in the mind’s eye’, or intrusive visual imagery – but should be experienced ‘inside the head’ or specifically distinguished from an external visual perception.

**A7. Does the participant report felt presences?**

Does the participant report feeling like someone or something is with them even when they can’t hear/see them? This does not have to be directly related to the voices.

**A8. Does the participant report tactile hallucinations?**

Includes descriptions of the participant feeling like someone or something is touching them. Does not have to be directly related to the voices. Note that this is separate from ‘bodily experiences’ (see below).

**A9. Does the participant report olfactory (smell) hallucinations?**

Descriptions of smelling things that are not actually present. Does not have to be directly related to the voices.

**A10. Does the participant report gustatory (taste) hallucinations?**

Descriptions of tasting things – does not have to be directly related to the voices.

**A11. Does the participant describe experiences that seem dissociative?**

“I just channel the message”, “I am not present/not aware when it goes on”, “I go into a kind of trance”, “My body feels inhabited by a spirit”, “I am a bit like a puppet”, “Afterwards I am tired/in a daze”, any mentions of memory loss.

**A12. Does the participant report any kind of change in bodily state?**

i.e., does the participant report experiences in their body – note that this is separate from ‘tactile’ experiences (above) in that it may include physical ‘symptoms’ like elevated heart rate, feelings like their body being controlled by others, feelings of warmth throughout the body, unusual interoceptive awareness etc.

1. **SPATIALITY**

**B1. Does the participant report internal voices?**

Does the participant report voices that feel like they are coming from inside the head or body? Note that this is not necessarily the same thing as ‘thought-like’ voices; for example, a person could describe a voice coming from the centre of their head that has clear ‘auditory’ properties.

**B2. Does the participant report external voices?**

Does the participant report voices that feel like they are coming from outside the head or body? For example, these could feel like they are located just next to the ear, or across the room etc. Note that an externally located voice could still be coded as ‘thought-like’, if it lacks auditory qualities and the participant describes it more with reference to/as being confused with thoughts.

**B3. Does the participant report ‘boundary voices’?**

The voice is experienced as ‘just beyond perception’, just out of earshot. For example, voices coming from just outside the room or house, through the walls, ‘round the corner’.

**B4. Does the participant report ‘egocentric’ voices?**

The voice is experienced as originating/emanating from a position relative to the participant – for example, ‘next to my ear’, ‘behind my head’.

**B5. Does the participant report ‘allocentric’ voices?**

The voice is experienced as originating/emanating from a position relative to surroundings, not the person – for example, ‘through the wall’, ‘from the next room’, or from an object nearby.

1. **ONSET**

**C1. Does the participant report traumatic events or context around time of first voice?**

Code if participants describe specifically difficult or stressful circumstances in months prior to or just after voice onset. May or may not be directly causally related by participant.

**C2. Can voices occur at participant’s own will/with volition?**

Does the participant describe being able to ‘tune in’ and bring the experience on intentionally, all or most of the time?

**C3. Does the participant report voices that occur without volition?**

Explicit mentions of the voice being outside control; e.g., how do I know it's not my thoughts – because I can't control it/them. “Voice happens to me, I have no control over its/their existence, unlike thoughts I can’t control the voices”, “They just happen sometimes”

**C4. Can the participant influence the voice?**

‘Influencing the voice’ is defined here and below as the voice-hearer being able to change the attitude, behavior or content of their voices, even if this is only temporary. The change must be voice-hearer initiated in some sense, but does not necessarily have to be achieved through conversation or felt to be beneficial. For example, Alex: “I’ll have a big conversation back…

So sometimes they’ll ease it off, sometimes it allows us to manage it better…Otherwise it

comes back a bit more intense and makes the situation slightly worser.”

**C5. Has the participant’s ability to influence the experience changed over time?**

Does the participant report getting better (or worse) at controlling the onset or offset of the experience over time?

**C6. Have the experiences got more or less frequent over time?**

Any reports of voice frequency increasing or decreasing over time. Frequency here refers to how many times a day/week any voices talk (not to an increase in the number of voices).

**C7. Is there evidence of structural longitudinal change?**

Reports of changes in: number of different identifiable voices, different voices coming or going, changes in ‘physical properties’ of voices, e.g. loudness, over time. Structural longitudinal change does not refer to ‘psychological’ or ‘characterful’ changes.

**C8. Is there evidence of voice character change?**

Reports of changes in the experience of specific voices. For example, changes in voice ‘personality’ or other psychological characteristics of the voice which are in some way sustained over time. For example, a voice could have gotten more ‘nasty’ with time, or become more ‘omnipotent’. “Physical” changes alone are not sufficient for code, although changes to C7 and C8 may coccur: “*he got quieter*…*was less mean*”.

1. **CHARACTERS AND CONTENT**

**D1. Does the participant report recurring voices?**

Does the person hear the same voice on different occasions? The importance here is the participant identifying it as the same voice (i.e., they could say it sounds different on separate occasions, but is the ‘same voice’).

**D2. Does the participant report recognizable voices?**

That is, does the participant recognize the voice from ‘real life’? Code ‘yes’ if the voice is of someone in the ‘real world’. Note it is not enough to just ‘remind’ the participant of a real person.

**D3. Do the voices elicit positive emotions?**

Does the participant describe feeling better due to the voices (e.g., the voices make them happy, feel better, offer companionship, or other positive emotions).

**D4. Do the voices elicit negative emotions?**

Does the participant describe feeling worse due to the voices (e.g., the voices make them feel sad, depressed, angry, or other negative emotions).

**D4a. Are the voices associated with anxiety?**

Subcode of D4. Do the voices prompt specific feelings of anxiety or worry, or can they be elicited or exacerbated by anxious states? Need to be able to evidence through specific reference, behaviour or context. If in doubt, don’t choose between D4 codes but apply all which are relevant.

**D4b. Are the voices associated with depression?**

Subcode of D4. Do the voices prompt specific feelings of depression or low mood, or can they be elicited or exacerbated by depressive states? Need to be able to evidence through specific reference, behaviour or context. If in doubt, don’t choose between D4 codes but apply all which are relevant.

**D4c. Are the voices associated with fear?**

Subcode of D4. Do the voices prompt specific feelings of fear or dread, or can they be elicited or exacerbated by fearful states? This code should pick up specific references to being afraid, scared, feeling of being threatened or that really bad things will happen in the future etc (more than anxious or worried).

**D4d. Are the voices associated with paranoia?**

Subcode of D4. Do the voices prompt specific feelings of paranoia or distrust, or can they be elicited or exacerbated by paranoid states?

**D5. Does the participant report voices with only a ‘simple’ structure?**

Voices consisting of single words or simple phrases. Refers to verbal not sound-only voices. Code ‘yes’ if the participant reports a voice that mainly (or solely) uses simple structure. Note that the participant could have other voices without a simple structure and still be coded ‘yes’.

**D6. Are the voices described as directly addressing the individual?**

Voices described as talking to the participant, i.e., using second person address (as opposed to descriptions of overhearing conversations, or voices talking about them).

**D7. Does the participant report commenting voices?**

Voices comment on actions or thoughts in the present moment. (This is not about voices “keeping up a running commentary” in the Schneiderian sense, or about the commentary being sustained, or in the third person.) For example, Alex: “Yes, certainly, just contradicting or commenting on things I’ve been doing around the house, as I’m doing them, as if someone’s actually there, watching you at the time they’re commenting, you know.”

**D8. Does the participant report voices which can be conversed with?**

Participant can have a conversation or dialogue with the voice, participant can and does ‘talk back’.

**D9. Does the participant report commanding voices?**

Voices command or instruct the participant to do things. This can include any form of instructions (for example, these could be quite benign (‘look under the sofa’), or commands to harm self).

**D10. Does the participant report abusive and/or violent voices?**

Voices are hostile, and may threaten or insult the participant, in the content of what they say.

**D11. Does the participant report positive and/or helpful voices?**

Voices are friendly, helpful, positive in the content of what they say.

**D12. Does the participant report getting companionship from the voice?**

Reports of the voices providing friendship/companionship.

**D14. Does the voice know things that the participant does not?**

The participant feels that the voice has knowledge that they do not (e.g., about events, other people, the future).

1. **LEVELS OF AGENCY**

**E1. Reports of voices with absent agency**

Some examples: hallucinated screams and groans, hearing ‘the last word or section of a phrase echoed’, hearing murmurs, hearing crowds. Mostly non-verbal; simple structure. For example, Dan: “sometimes I will hear stuff like crashing, explosions and things like that, along with the screaming, if that makes sense.” NOT “enemy footsteps,” which would be level 2.

**E2. Agency without individuation**

Agency is represented but without a distinguishable agent. Hypervigilance hallucinations may often fall into this category. ‘It is clear that there is a communicative intention and that the subject takes the experienced utterance to be directed at him’. Examples: hearing insults shouted at them (but not from anyone in particular). For example, Carl: “it’s like, it taps, and it ends up getting louder and louder, but I can feel that, I can actually feel them tapping, like wherever it is I can feel it, and it gets worse and worse and worse.”

**E3. Internally individualized agency**

‘Voices associated with agents identifiable by individual characteristics, but without any nominative reference’. Examples: unknown old woman; man with a deep voice. Voices may also be given ‘an idiosyncratic or purely internally referenced name, for example ‘the demonic’, ‘Simon’.

**E4. Externally individuated agency**

Voices associated with specific identities from the outside world (but more than just reminding the voice-hearer of someone). Could include past or current family members, acquaintances or celebrities, or fictional/non-corporeal individuals from popular culture or religion.

**E5a Minimal Personification**

Voices are described as having a single person-like quality, or are described as being “like a person” without further details being provided, or ascribed to a person without further elaboration. Complexity should be evidenced (see below) not inferred – voices with named identities (‘Simon’, ‘my mother’) can still be minimally personified (contra Wilkinson and Bell).

**E5b Complex Personification**

Voices are described as having more than one kind of person-like quality. These may include descriptions of intentional states (the voice wants/thinks/feels), descriptions of agency (the voice will “make something happen” if I do X), or elaborate descriptions of identity (the voice “comes” from somewhere, the voice has a specific and idiosyncratic ontological status, the voice is known). Complexity is not a simple function of frequency, quantity or topic, but it is likely that voices which frequently speak about a variety of aspects of the world or the voice-hearers’ life will have more complex personification than voices which appear sporadically, or have a narrow focus. Similarly voices which appear in more than one sensory modality.

**E5c Archetypal Features**

Voices are described as taking stereotypical forms from myth or culture, i.e. identities that recur in common stories, folk tales, religious or spiritual texts. Can be spiritual entities (angels, demons etc) as well as gender/social stereotypes (the angry or aggressive man, the kind old lady, the frightened child).

1. **SOCIAL CONTEXT AND INTERPRETATION**

**F1. Are the voices important to the participant’s sense of identity/sense of self?**

Are the voices talked about as an important part of the participant’s identity? For example, would they miss them if they were gone/would they feel like they were missing a part of themselves?

**F2. Does the participant describe the voices as having had a positive impact on relationships with others?**

Does the participant describe having voices as strengthening/making better, relationships with others?

**F3. Does the participant describe the voices as having had a negative impact on relationships with others?**

Does the participant describe having voices as having negatively affected relationships with others, e.g., causing ends of friendships or close relationships, or drawing away from others due to voices.

**F4. Does the participant self-stigmatise?**

For example – I wouldn’t tell others because they would think I was mad; ‘voices mean I’m crazy’.

**F5. Presence of a biophysical narrative**

Participants attributes the voices to biological causes, e.g., something going on with my brain, or reference to voices being caused by physical health issues, sleep, illness, drugs, alcohol, etc. Participant has to posit a causal link of some kind but may also have other explanations.

**F6. Presence of a trauma narrative**

Participant attributes the voices to past severe or adverse events, e.g., emotional, physical, or sexual abuse, witness of or victim to violence, serious accidents or injuries, combat experience, sustained bullying, sudden bereavement.

**F7. Presence of a stress narrative**

Participant attributes the voices to a stressful event or period of sustained stress reflecting mild or moderate adverse life events, e.g. changes in education, job, housing, family relationships.

**F8: Idiosyncratic narrative**

Participant explains voices in own narrative (i.e., that may or may not only pertain to them). For example, they may believe they are being spied on or persecuted, or the voice belongs to a real person who is ‘in their head’. For example, Eric: “obviously invented them because I needed them, ehm, or they’ve kind of gone on from maybe I had an overactive imagination.”

**F9. Presence of a supernatural/spiritual narrative**

Participant explains the voices using supernatural or spiritual terms (e.g., believes they are voices of dead people, believes they are a real entity communicating using telepathy).

**F10. Presence of a ‘family’ narrative**

Participant believes that their voices are ‘in the family’ (e.g., they mention a family member having voices/psychosis and link their voices to that; mention voices being ‘in their genes’).

**F11. Does the participant report sleep disruption?**

Any mention of the participant being unable to sleep or having unusual sleep patterns.

**F12. Does the participant report suicidal thoughts?**

Any mention of the participant showing suicidal ideation, or mention of actual suicide attempts. Does not necessarily have to be in direct connection with voices.

**(Spiritualist only codes)**

**F18-SPI. Was the first experience of voices spontaneous, or aspirational?**

Did the experience ‘just start’ with no intentional development by the participant, or did the participant deliberately develop them from scratch (e.g., by attending a spiritualist church)?

**F19-SPI. Did the participant hear voices before becoming a spiritualist?**

Does the participant report voice experiences before becoming a spiritualist? (Voices may or may not have led them to approach a spiritualist church.)

**F20-SPI. Does the participant work in a ‘professional’ capacity with the voices?**

Descriptions of ‘working’, for example as a medium or psychic. (Payment not a prerequisite for a ‘yes’ code.)

**F21-SPI. Does the participant view voices as a skill that can be learnt by anyone?**

Code ‘yes’ if the participant describes a view that anyone (or almost anyone) could learn clairaudience/voices; code ‘no’ if the participant describes the view that some people have a ‘gift’ that others don’t (or describes another viewpoint in which not anyone could hear voices). Note that this can be coded ‘yes’ if the participant believes some people are naturally better than others, as long as they state that anyone could, technically, learn the skill.

**F22-SPI. Does the participant deliberately ‘practice’ or cultivate their voices?**

Does the participant describe regularly having to practice or develop voices to ensure they can hear or control them?

**F23-SPI. Is the participant completely convinced that the voices are spirits/fit within a spiritualist framework?**

Code ‘yes’ if the participant shows no doubt about the ‘reality’ of the voices as spirits, or voices of the dead/from another world etc. Code ‘no’ if the participant is at all unsure of this framework.

**Further details and examples from the coding frame can be found here:** [**https://osf.io/bd2uy/**](https://osf.io/bd2uy/)

**S3. Age of onset, and time since onset of voices**

We did not explicitly gather information on time since onset of voices. However, participants were asked about the age of onset, as well as providing demographic information including their age. Information regarding participant age can be found in the Method section of the manuscript.

The spiritual group reported voice onset at a younger age (M = 16.90, SD = 14.45) than the patient group (M = 22.68, SD = 13.45), although this was not statistically significant (U = 358, p = .076, r = .27).

We calculated ‘time since onset (years)’ by subtracting estimated age of onset from participants’ age at participation. There was a large difference between the time since onset between the groups, with large variation and ranges within both groups. In the patient group, the mean time since onset (years) was 6.59 (SD = 7.3, range = 0-27). In the spiritual group, the mean time since onset was 41.98 (SD = 17.2, range = 8-71). This difference was statistically significant (U = 26.5, p < .001, r = .95).

This discrepancy likely reflects a number of factors. Firstly, it reflects the way in which the patient group was recruited. That is, the study for which they were recruited specifically tried to recruit participants soon after they first approached mental health services. There was no such recruitment constraint for the spiritual group. Secondly, the older age of the spiritual group probably reflects a core attribute of this group – that is, the group we tried to recruit, and were interested in interviewing, tend to be older adults. Many had heard voices for a long period of time, but others were older at initial voice onset. Thirdly, the spiritual group reported a somewhat earlier age of onset than the patient group (as has been reported in a number of other studies of non-clinical voice-hearers), further increasing the time since voice onset.

As noted in the manuscript, there are a number of differences between the patient group and the spiritual group. The groups were not deliberately matched on any attributes because the two samples were collected as part of different studies. Attributes such as age, gender, clinical status, and time since voice onset likely interact in a number of ways that is beyond the scope of the present study. Here, we present the patient group mainly as a ‘reference’ group (i.e., a baseline against which to contextualise the data from the spiritualist group).

**S4: mean scores for PSYRATS subscales in spiritual voice-hearers and voice-hearing**

| **PSYRATS subcale** | **Spiritual voice-hearers**  **(M, SD)** | **Patients**  **(M, SD)** |
| --- | --- | --- |
| Physical | 5.31 (2.87) | 9.32 (2.20) |
| Cognitive | 3.92 (2.28) | 6.50 (1.95) |
| Emotional | 0.08 (0.39) | 9.95 (4.11) |
| AH overall | 9.54 (4.25) | 25.78 (6.28) |

**psychosis patients**
